# Cryptochrome-mediated blue-light signalling modulates UVR8 photoreceptor activity and contributes to UV-B tolerance in Arabidopsis

**DOI:** 10.1038/s41467-020-15133-y

**Published:** 2020-03-12

**Authors:** Nicolas Tissot, Roman Ulm

**Affiliations:** 1 Department of Botany and Plant Biology, Section of Biology, Faculty of Sciences, University of Geneva, 30 Quai E. Ansermet, 1211 Geneva 4, Switzerland; 2 Institute of Genetics and Genomics of Geneva (iGE3), University of Geneva, Geneva, Switzerland

**Keywords:** Plant sciences, Light responses, Plant signalling, Plant stress responses

## Abstract

UV-B constitutes a critical part of the sunlight reaching the earth surface. The homodimeric plant UV-B photoreceptor UV RESISTANCE LOCUS 8 (UVR8) monomerizes in response to UV-B and induces photomorphogenic responses, including UV-B acclimation and tolerance. REPRESSOR OF UV-B PHOTOMORPHOGENESIS 1 (RUP1) and RUP2 are negative feedback regulators that operate by facilitating UVR8 ground state reversion through re-dimerization. Here we show that *RUP1* and *RUP2* are transcriptionally induced by cryptochrome photoreceptors in response to blue light, which is dependent on the bZIP transcriptional regulator ELONGATED HYPOCOTYL 5 (HY5). Elevated RUP1 and RUP2 levels under blue light enhance UVR8 re-dimerization, thereby negatively regulating UVR8 signalling and providing photoreceptor pathway cross-regulation in a polychromatic light environment, as is the case in nature. We further show that cryptochrome 1, as well as the red-light photoreceptor phytochrome B, contribute to UV-B tolerance redundantly with UVR8. Thus, photoreceptors for both visible light and UV-B regulate UV-B tolerance through an intricate interplay allowing the integration of diverse sunlight signals.

## Abstract (web summary)

The Arabidopsis UVR8 photoreceptor is a dimer that monomerizes in response to UV-B. Here the authors show that cryptochromes contribute to UV tolerance and facilitate UVR8 redimerization via induction of RUP proteins in response to blue light, modifying UV-B signalling in polychromatic light environments.

## Introduction

Sunlight is an essential factor for life on earth. It is the ultimate source of energy for photosynthesis and it modulates plant growth and development. Flowering plants can perceive light signals spanning from UV-B to far-red (~280–800 nm) using five classes of photoreceptors^1^. UV-B (280–315 nm) induces photomorphogenic responses and UV-B acclimation^2–5^. The UV-B photoreceptor UV RESISTANCE LOCUS 8 (UVR8) forms homodimers in its ground state and monomerizes to the active form in response to UV-B absorption^6,7^. UVR8 monomers interact directly with the WD40 domain of the E3 ubiquitin ligase CONSTITUTIVELY PHOTOMORPHOGENIC 1 (COP1)^5,7^. The UVR8−COP1 interaction competes with that between COP1 and the photomorphogenesis-promoting bZIP transcription factor ELONGATED HYPOCOTYL 5 (HY5), thereby preventing its ubiquitination^8,9^. This leads to stabilization of HY5 and the induction of many target genes encoding proteins involved in the photomorphogenic response to UV-B, including HY5 itself^10–12^. Among these HY5-induced genes, *REPRESSOR OF UV-B PHOTOMORPHOGENESIS 1* (*RUP1*) and *RUP2* encode WD40-repeat proteins that provide UVR8 negative feedback regulation^13^. RUP1 and RUP2 directly interact with UVR8 to facilitate its re-dimerization, thereby inactivating the UVR8 monomer^14,15^. RUP1 and RUP2 can also be part of a CUL4-DDB1-based E3 ubiquitin ligase that targets HY5 for degradation^16^. Moreover, it has been proposed that COP1 directly targets RUP1 and RUP2 for ubiquitination and degradation under UV-B, contributing to the stabilization of HY5^16^.

Cryptochrome blue-light signalling shows some interesting similarities to UVR8 UV-B signalling. The oligomeric state of cryptochromes changes in response to blue-light perception, specifically from an inactive monomeric to an active homodimeric state^17^. BLUE-LIGHT INHIBITOR OF CRYPTOCHROMES (BIC1) and BIC2 provide negative feedback regulation by directly binding to cryptochromes and inhibiting their dimerization^17,18^. Finally, active cryptochromes also inhibit the COP1 E3 ubiquitin ligase complex, which results in HY5 stabilization and accumulation^9,19–24^. Moreover, synergisms and interplays between cryptochrome and UV-B/UVR8 signalling have been described before; however, these remain poorly understood at the molecular level^25–28^.

Here, we show that induction of *RUP1* and *RUP2* gene expression and their ensuing protein accumulation are blue-light responsive. These inductions depend mainly on the blue-light photoreceptor cry1, through the activity of HY5, with lesser roles played by cry2 and phyA. Enhanced RUP1 and RUP2 levels under blue light affect the balance between UVR8 monomer and UVR8 homodimer, thereby modulating the activity of the UV-B signalling pathway. Finally, we demonstrate that cry1, phyB, and UVR8 redundantly regulate UV-B tolerance.

## Results

### Cryptochromes and phyA activate *RUP1* and *RUP2* expression

**Fig. 1:**
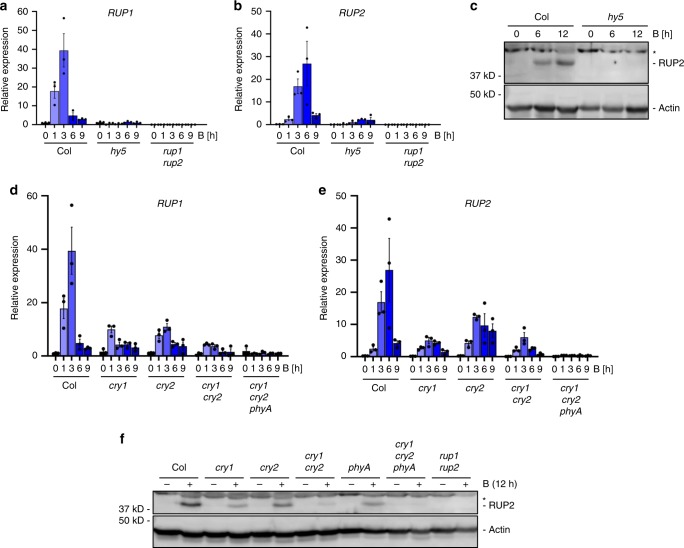
Blue-light-induced *RUP1* and *RUP2* expression and RUP2 protein accumulation depend on cry1, cry2, phyA, and HY5. **a**, **b** qRT-PCR analysis of **a** *RUP1* and **b** *RUP2* expression in 4-d-old wild type (Col), *hy5-215* (*hy5*), and *rup1-1 rup2-1* (*rup1 rup2*) seedlings grown in darkness or treated with blue light (B; 50 µmol m^−2^ s^−1^) for 1, 3, 6, or 9 h. Data are means ± SEM (*N* = 3). **c** Immunoblot analysis of RUP2 protein level in 4-d-old Col and *hy5* seedlings grown in darkness (0) or treated with blue light for 6 or 12 h. The asterisk indicates a nonspecific cross-reacting band. Actin is shown as protein loading control. **d**, **e** qRT-PCR analysis of **d** *RUP1* and **e** *RUP2* expression in 4-d-old Col, *cry1-304* (*cry1*), *cry2-1* (*cry2*), *cry1-304 cry2-1* (*cry1 cry2*), and *cry1-304 cry2-1 phyA-412* (*cry1 cry2 phyA*) seedlings grown in darkness or treated with blue light for 1, 3, 6, or 9 h. Data are means ± SEM (*N* = 3). Note that the Col samples in panels (**d**) and (**e**) are identical to those in panels (**a**) and (**b**), respectively, as the data are derived from the same experiment. **f** Immunoblot analysis of RUP2 protein level in 4-d-old Col, *cry1*, *cry2*, *cry1 cry2*, *phyA-211* (*phyA*), *cry1 cry2 phyA*, and *rup1 rup2* seedlings grown in darkness, then treated with blue light for 12 h (+) or not (−). The asterisk indicates a nonspecific cross-reacting band. Actin is shown as protein loading control.

Blue-light exposure of Arabidopsis seedlings resulted in strong and transient induction of *RUP1* and *RUP2* expression in wild type, but not in *hy5* (Fig. 1a, b). In agreement, RUP2 protein accumulated in response to blue light in wild type, but not in *hy5* to a detectable level (Fig. 1c). To identify the photoreceptors responsible for the blue-light induction of *RUP1* and *RUP2* expression, we examined responses in *cry1*, *cry2*, *cry1 cry2*, and *cry1 cry2 phyA*. *cry1* and *cry2* single mutants both displayed reduced blue-light induction of *RUP1* and *RUP2*, which was further reduced in *cry1 cry2* double mutants and absent in *cry1 cry2 phyA* triple mutants (Fig. 1d, e). In agreement, RUP2 protein accumulation in response to blue light was reduced in *cry1*, *cry2*, and *phyA*, further reduced in *cry1 cry2*, and undetectable in *cry1 cry2 phyA* (Fig. 1f). The absence of an anti-RUP1 antibody prevented directly testing endogenous RUP1 levels. We conclude that blue-light-dependent cryptochrome and phyA signalling activates *RUP1* and *RUP2* expression, resulting in RUP2, and likely RUP1, protein accumulation.

### cry1 and phyA signalling enhances UVR8 re-dimerization

**Fig. 2:**
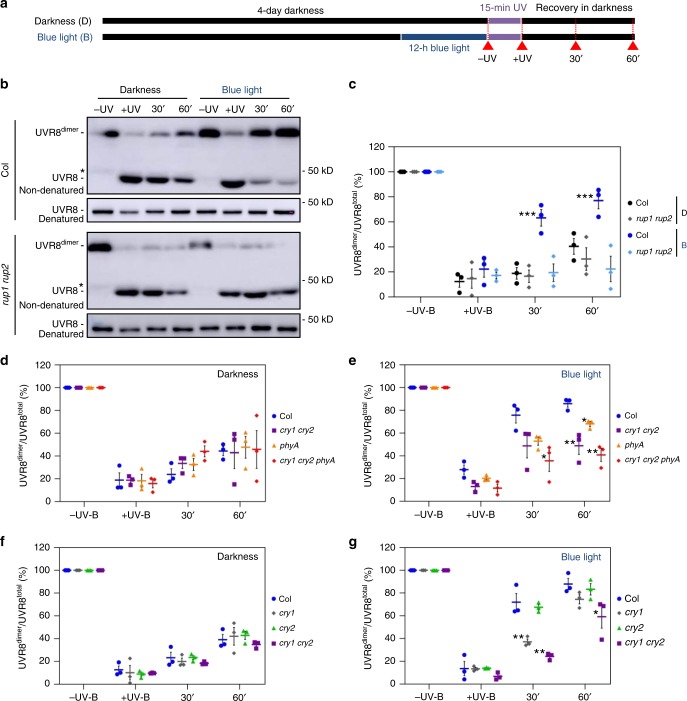
cry1 and phyA are the main photoreceptors mediating the RUP1- and RUP2-dependent blue-light repression of UVR8 activity. **a** Schematic representation of the light treatments of seedlings for the UVR8 re-dimerization analyses. Four-d-old etiolated seedlings were exposed to 12-h blue light (B, 50 μmol m^−2^ s^−1^) or maintained in darkness (D) before they were irradiated for 15 min with UV-B (0.85 mW cm^−2^) and maintained further in darkness for 1 h. The time points of sampling are indicated by red arrows labelled with −UV-B, +UV-B, 30′, and 60′. **b** Immunoblot analysis of UVR8 re-dimerization in wild type (Col) and *rup1-1 rup2-1* (*rup1 rup2*) seedlings. UVR8 dimers were detectable in nonheat-denatured protein samples. Parallel denatured samples demonstrate equal amounts of UVR8 protein. The asterisk indicates a nonspecific cross-reacting band. **c** Quantification of the UVR8^dimer^/UVR8^total^ ratio (%) in Col and *rup1 rup2*. **d**, **e** Quantification of the UVR8^dimer^/UVR8^total^ ratio (%) in response to UV-B in Col, *cry1-304 cry2-1* (*cry1 cry2*), *phyA-211* (*phyA*), and *cry1-304 cry2-1 phyA-412* (*cry1 cry2 phyA*) seedlings pre-treated with blue light (**e**) or not (**d**). **f**, **g** Quantification of the UVR8^dimer^/UVR8^total^ ratio (%) in response to UV-B in Col, *cry1-304* (*cry1*), *cry2-1* (*cry2*), and *cry1 cry2* seedlings pre-treated with blue light (**g**) or not (**f**). Data are means ± SEM (*N* = 3). *P < 0.05, **P < 0.01, and ***P < 0.001.

Accumulation of RUP1 and RUP2 in response to blue light points to a previously unknown effect of blue-light signalling on UVR8 activity. We thus tested the effect of blue light on the dynamics of the UVR8 homodimer/monomer ratio upon UV-B treatment, with a particular focus on UVR8 re-dimerization post UV-B exposure (Fig. 2a). The UV-B treatment induced a strong UVR8 monomerization in wild type and *rup1 rup2* but did not affect the total amount of UVR8 (−UV and +UV; Fig. 2b, c). During the subsequent recovery in darkness, UVR8 re-dimerization was significantly faster in wild-type seedlings that were pre-exposed to blue light than that in seedlings without blue-light treatment (30′ and 60′; Fig. 2b, c). This blue-light enhancement of UVR8 re-dimerization was absent in *rup1 rup2* mutants, in which UVR8 remained monomeric during the 1-h post-UV-B recovery (Fig. 2b, c). UVR8 re-dimerization was also impaired in *rup1* and *rup2* single mutants but to a considerably lesser extent than in the *rup1 rup2* double mutant, supporting redundant roles of RUP1 and RUP2 in this process (Supplementary Fig. 1)^13,14^. Moreover, in agreement with the role of HY5 in *RUP1* and *RUP2* activation, blue-light-enhanced UVR8 re-dimerization was strongly impaired in *hy5* mutants (Supplementary Fig. 2).

To test the roles of specific photoreceptors in blue-light-enhanced UVR8 re-dimerization, we quantified the UVR8^dimer^/UVR8^total^ ratio in wild-type seedlings and *cry1*, *cry2*, *cry1 cry2*, *phyA*, and *cry1 cry2 phyA* mutant seedlings. Without blue-light pre-treatment, no differences were observed in UVR8 re-dimerization kinetics among the six tested genotypes (Fig. 2d, f). However, in blue-light pre-treated seedlings, the enhanced UVR8 re-dimerization apparent in wild type was partially impaired in *phyA* and considerably more so in *cry1 cry2* and *cry1 cry2 phyA* (Fig. 2e). This result agrees with the reduced RUP2 accumulation in response to blue light observed in these photoreceptor mutant lines (Fig. 1e). In further agreement with a key role of cry1, overexpression of GFP-cry1 resulted in elevated levels of RUP2 in response to blue light and further enhanced UVR8 re-dimerization when compared to wild type under these conditions (Supplementary Fig. 3). In contrast to *cry1* and *cry1 cry2*, blue-light pre-treated *cry2* single mutants displayed UVR8 re-dimerization kinetics comparable to that in wild type (Fig. 2g). We conclude that blue light enhances UVR8 re-dimerization, and that this is mainly due to cry1 and phyA signalling-mediated RUP1 and RUP2 accumulation.

### BIC overexpression blocks enhanced UVR8 re-dimerization

**Fig. 3:**
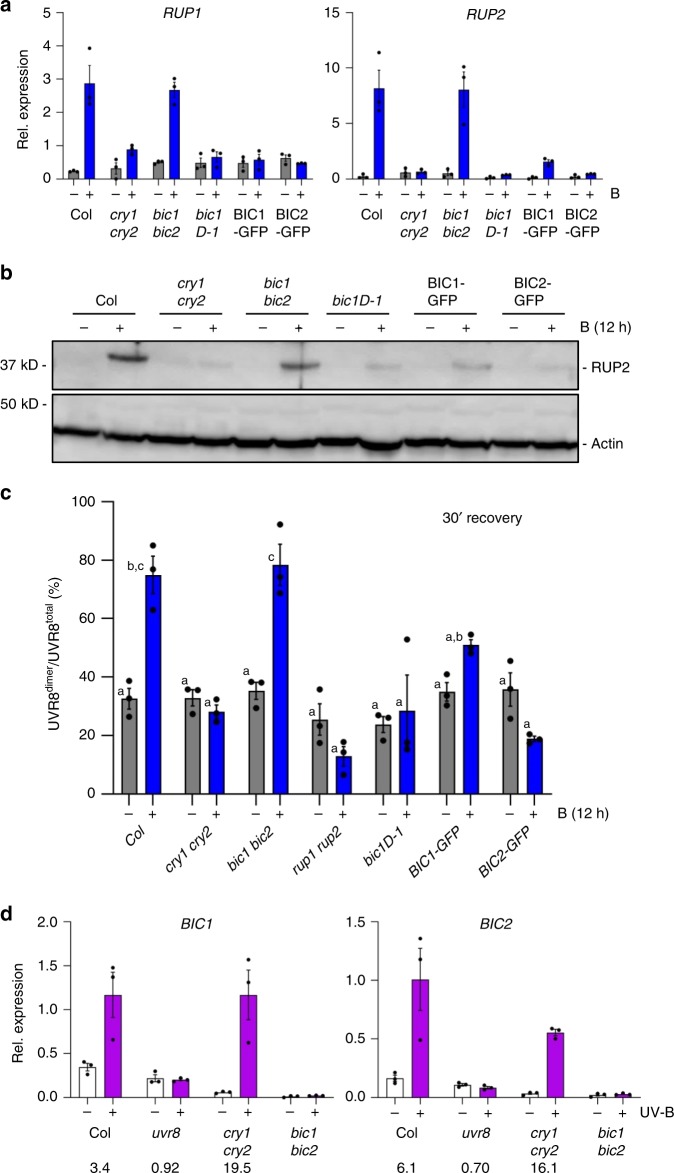
BIC1 and BIC2 repress blue-light-induced RUP2 accumulation and UVR8 re-dimerization. **a** qRT-PCR analysis of *RUP1* (left panel) and *RUP2* (right panel) mRNA levels in 4-d-old Col, *cry1 cry2*, *bic1 bic2*, *Pro*_*35S*_*:BIC1* (*bic1D-1*), *Pro*_*35S*_*:BIC1-GFP* (BIC1-GFP), and *Pro*_*35S*_*:BIC2-GFP* (BIC2-GFP) seedlings grown in darkness and exposed to blue light (B; 50 μmol m^−2^ s^−1^) for 6 h (+) or not (−). Data are means ± SEM (*N* = 3). **b** Immunoblot analysis of RUP2 protein level in 4-d-old Col, *cry1 cry2*, *bic1 bic2*, *bic1-D1*, BIC1-GFP, and BIC2-GFP seedlings grown in darkness and exposed to 12 h of blue light (+) or not (−). Actin is shown as protein loading control. **c** Quantification of the UVR8^dimer^/UVR8^total^ ratio (%) after a 30-min recovery post-UV-B treatment in wild type (Col), *cry1 cry2*, *bic1 bic2*, *rup1 rup2*, *bic1D-1*, BIC1-GFP, and BIC2-GFP seedlings pre-treated with 12-h blue light (+) or not (−). Data are means ± SEM (*N* = 3). Shared letters indicate no statistically significant difference in the means (*P* > 0.05; one-way ANOVA followed by post-hoc Tukey test). **d** qRT-PCR analysis of *BIC1* (left panel) and *BIC2* (right panel) mRNA levels in 4-d-old wild type (Col), *uvr8-6* (*uvr8*), *cry1-304 cry2-1* (*cry1 cry2*), and *bic1-1 bic2-1* (*bic1 bic2*) seedlings grown in white light (3.6 μmol m^−2^ s^−1^) and exposed to UV-B for 3 h at 0.06 mW cm^−2^ (+) or not (−). Data are means ± SEM (*N* = 3); wild-type Col grown in white light without UV-B was set = 1. Numbers below the graphs show the fold change values under +UV-B compared with that under −UV-B for each genotype.

BIC1 and BIC2 are negative feedback regulators of cry1 and cry2^17^. We therefore tested whether BIC1 and BIC2 accumulation could affect *RUP1*/*RUP2* expression and RUP2 protein levels. Blue-light-induced accumulation of *RUP1*/*RUP2* mRNA and RUP2 protein was indeed reduced in the BIC1 and BIC2 overexpression lines *bic1-D1*, BIC1-GFP, and BIC2-GFP to a similar level as in *cry1 cry2* (Fig. 3a, b). This agrees with inhibitory roles of BIC1 and BIC2 in cry1 and cry2 activities^17^. However, we did not observe enhanced RUP2 protein levels in the *bic1 bic2* double mutant compared to that in wild type (Fig. 3b). To test whether BIC1 and BIC2 overexpression modulates UVR8 activity, we UV-B treated various genotypes that were pre-treated with blue light or not, and then quantified UVR8 re-dimerization after 30-min recovery. In agreement with the observed RUP2 levels, blue-light-enhanced UVR8 re-dimerization was similar in *bic1 bic2* and wild type, but strongly impaired in the BIC1 and BIC2 overexpression lines, comparable to that in *cry1 cry2* and *rup1 rup2* (Fig. 3c). Interestingly, reciprocally to *RUP1* and *RUP2* activation through cryptochrome-mediated blue-light signalling (Fig. 1)^13^, *BIC1* and *BIC2* are induced by UVR8-mediated UV-B signalling (Fig. 3d)^5^. We conclude that inhibition of cryptochrome activity by BIC1 and BIC2 suppresses the blue-light enhancement of UVR8 re-dimerization, and that UVR8-mediated regulation of *BIC1* and *BIC2* may add an additional regulatory control level.

### cry1 and cry2 repress UVR8-induced responses

**Fig. 4:**
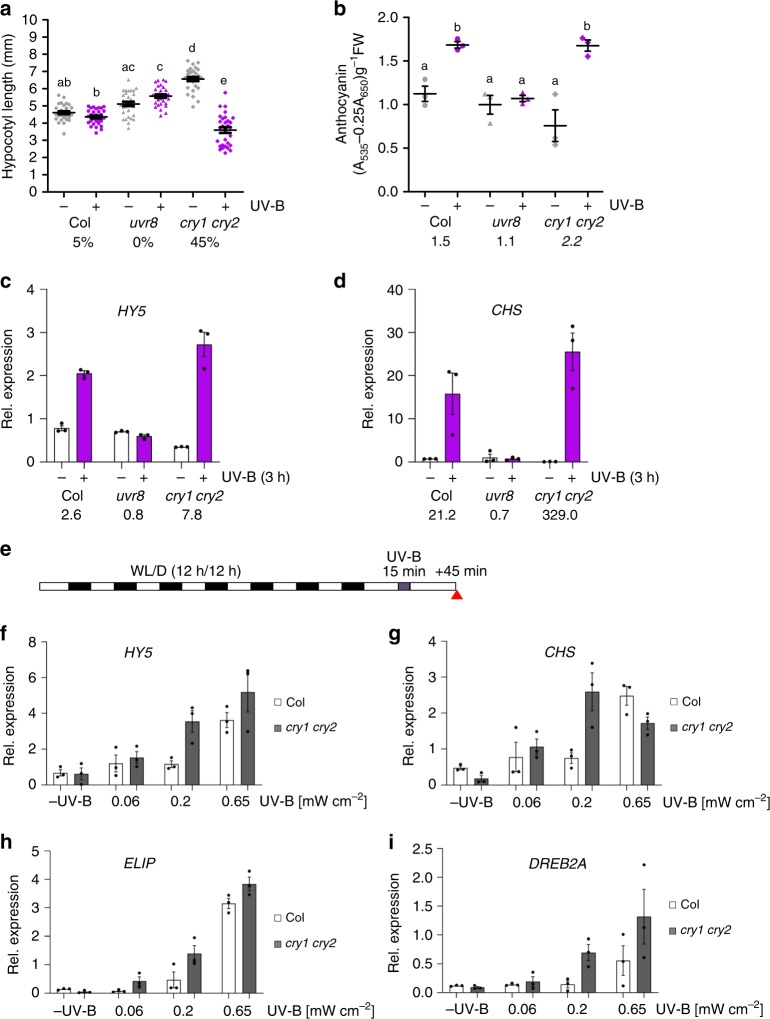
cry1 and cry2 repress UVR8-mediated UV-B responses. **a** Quantification of hypocotyl length in 4-d-old wild type (Col), *uvr8-6* (*uvr8*), and *cry1-304 cry2-1* (*cry1 cry2*) seedlings grown in white light (3.6 μmol m^−2^ s^−1^) supplemented with UV-B (+UV-B; 0.015 mW cm^−^^2^) or not (−UV-B). Data are means ± SD (*N* = 30). Shared letters indicate no statistically significant difference in the means (*P* > 0.05; one-way ANOVA followed by post-hoc Tukey test). Numbers below bars show the relative hypocotyl growth inhibition by UV-B as a percentage. **b** Quantification of anthocyanin accumulation. Data are means ± SEM (*N* = 3). Numbers below bars show the fold change values under +UV-B compared with that under −UV-B. **c**, **d** qRT-PCR analysis of **c** *HY5* and **d** *CHS* mRNA levels in 4-d-old Col, *uvr8*, and *cry1 cry2* seedlings grown in white light and exposed to UV-B at 0.06 mW cm^−^^2^ for 3 h (+) or not (−). Data are means ± SEM (*N* = 3); wild-type Col grown in white light without UV-B was set = 1. Numbers below bars show the fold change values under +UV-B compared with that under −UV-B for each genotype. **e** A schematic representation of the irradiation conditions. Seven-d-old seedling grown under 12-h light (WL)/12-h dark (D) cycles were irradiated for 15 min with broadband UV-B at different intensities (0.06, 0.20, and 0.65 mW cm^−2^) or not (−UV). The red arrow indicates when plant samples were harvested 45 min following the UV-B treatment; see also ref. ^12^. **f**−**i** qRT-PCR analysis of **f** *HY5*, **g** *CHS*, **h** *ELIP2*, and **i** *DREB2A* in Col and *cry1 cry2* seedlings grown as per the schematic in panel (**e**). Data are means ± SEM (*N* = 3).

The impact of blue-light signalling on UVR8 activity indicates an associated impact on UV-B responses. In comparison to wild type, *cry1 cry2* double mutants showed enhanced hypocotyl growth inhibition under supplementary UV-B (Supplementary Fig. 4a). It is of note, however, that *cry1 cry2* shows an elongated hypocotyl in the absence of UV-B compared to wild type, and a similar hypocotyl length in the presence of UV-B (Supplementary Fig. 4a). We thus reduced the UV-B to levels that were insufficient to induce hypocotyl growth inhibition in wild type to further test for UV-B hypersensitivity in *cry1 cry2*. Strikingly, *cry1 cry2* displayed a clear hypocotyl growth inhibition response under these conditions (Fig. 4a). This was further confirmed to be the case for a *cry1* mutant in the Ws accession (Supplementary Fig. 4b). A minor difference was also observed in UV-B-responsive anthocyanin accumulation between wild type and *cry1 cry2* under these conditions (Fig. 4b). We further investigated expression of UV-B-responsive genes. We first compared the induction of *HY5* and *CHALCONE SYNTHASE* (*CHS)* in *cry1 cry2*, *uvr8*, and wild type in response to 3-h supplemental narrowband UV-B^5,29^. Whereas *uvr8* mutants did not respond to UV-B, the *cry1 cry2* double mutant showed an enhanced activation of *HY5* (7.8-fold) and *CHS* (329.5-fold) expression in response to UV-B in comparison to that in wild type (2.2- and 21.2-fold, respectively) (Fig. 4c, d, respectively). We further analysed the effect of cry1 and cry2 on UVR8-mediated marker gene expression in response to treatment with 15-min broadband UV-B at three different irradiation levels followed by 45 min in white light (Fig. 4e). These UV-B irradiation conditions have previously been shown to induce expression of *HY5*, *CHS*, *EARLY LIGHT-INDUCIBLE PROTEIN 2* (*ELIP2*), and *DEHYDRATION-RESPONSIVE ELEMENT BINDING PROTEIN 2A* (*DREB2A*) in a COP1- and UVR8-dependent manner^5,12,29^. Although the response of the GFP-cry1 overexpression line resembled wild type (Supplementary Fig. 3c−f), the four tested genes showed consistently enhanced UV-B-induced expression in *cry1 cry2* compared to wild type, in further agreement with UVR8 hyperactivity in *cry1 cry2* (Fig. 4f−i). Our data from various distinct UV-B irradiation conditions indicate that cryptochrome signalling represses UVR8-mediated transcriptional responses.

### cry1 and phyB establish UV-B tolerance alongside UVR8

**Fig. 5:**
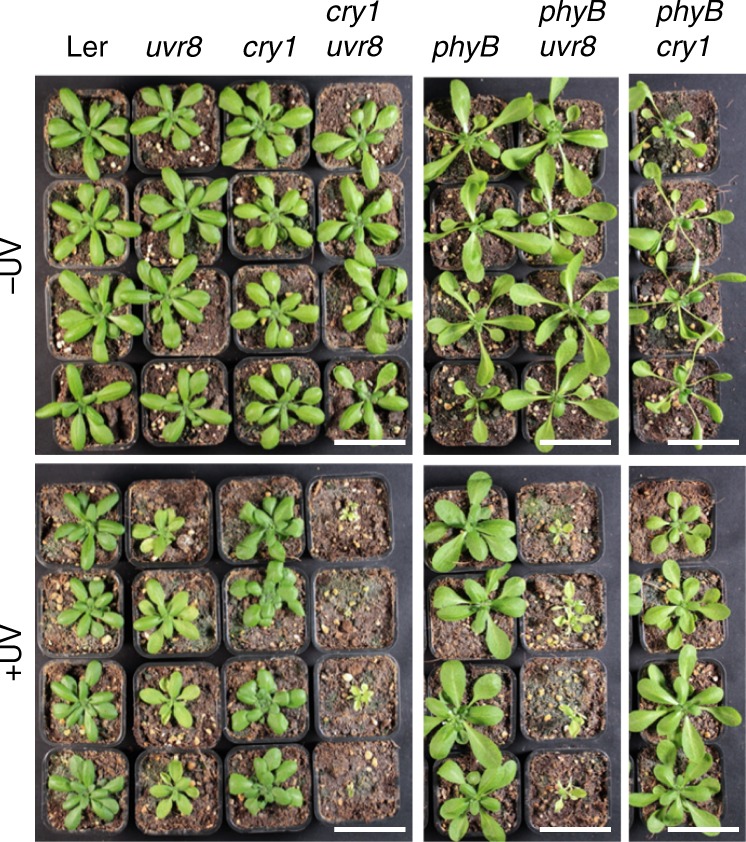
Cryptochromes and phytochromes redundantly contribute to UVR8-mediated UV-B tolerance. Photographs of 28-d-old wild-type (Ler), *uvr8-1* (*uvr8*), *hy4-2.23N* (*cry1*), *hy4-2.23N uvr8-1* (*cry1 uvr8*), *phyB-5* (*phyB*), *phyB-5 uvr8-1* (*phyB uvr8*), and *phyB-5 hy4-2.23N* (*phyB cry1*) plants grown under long-day conditions with 120 µmol m^−2^ s^−1^ of white light with supplemental UV-B (+UV, 0.07 mW cm^−^^2^) or not (−UV). Bars = 5 cm.

Cryptochrome blue-light and phytochrome red-light signalling are known to induce expression of genes that largely overlap with UVR8-induced genes. These co-induced genes include those associated with UV-B tolerance, such as *CHS* and *FLS*; those encoding phenylpropanoid biosynthetic enzymes, which are important for the synthesis of flavonol glycosides that function as sunscreen metabolites; and *PHR1* and *UVR3*, which encode photolyases important for DNA damage repair^5,17,30^. This indicates that, in addition to cry1 negative regulation of UVR8 activity, cry1 as well as phyB may contribute to UV-B tolerance alongside UVR8. In the absence of UV-B, the viability of *cry1*, *phyB*, and *uvr8* single mutants and *cry1 uvr8*, *phyB uvr8*, and *phyB cry1* double mutants was not affected (Fig. 5). The main observable growth difference without UV-B was the long petioles of *phyB* plants that display a constitutive shade-response phenotype (Fig. 5), as described before^31,32^. In the presence of UV-B, *cry1* and wild-type plants showed comparable growth reductions (Fig. 5). In addition to the smaller rosette phenotype under UV-B, *uvr8* plants displayed chlorotic leaves but remained viable (Fig. 5). Most strikingly, however, we observed that *cry1 uvr8* plants were extremely UV-B hypersensitive and did not survive under UV-B irradiation (Fig. 5). The constitutive shade-response phenotype of *phyB* evident without UV-B was clearly suppressed in the presence of UV-B (Fig. 5), in agreement with the finding that UVR8 antagonizes shade-avoidance responses^33^. *phyB uvr8* plant survival was also clearly impaired under UV-B, although *phyB uvr8* plants were less UV-B sensitive than *cry1 uvr8* (Fig. 5). Interestingly, in stark contrast to *cry1 uvr8* and *phyB uvr8* plants, *cry1 phyB* double-mutant plants maintained UV-B tolerance (Fig. 5). We conclude that, in addition to the major role UVR8 plays in establishing UV-B acclimation and tolerance, blue light-activated cry1 and red light-activated phyB contribute to full UV-B tolerance.

## Discussion

**Fig. 6:**
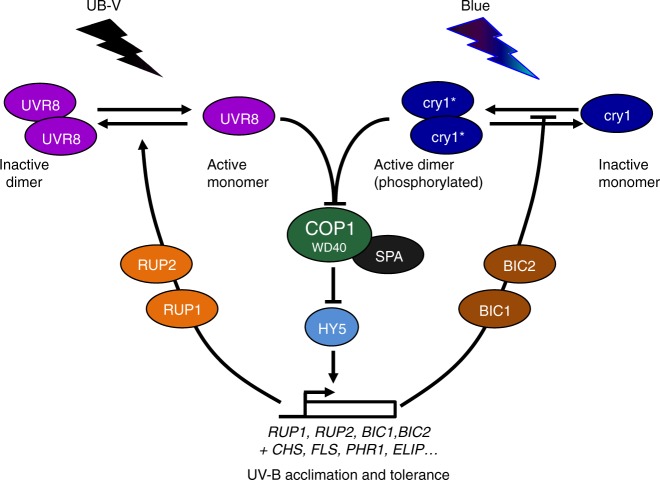
Working model for the molecular integration of UVR8 and cryptochrome signalling. Light activation of UVR8 and cry1 leads to inhibition of the COP1-SPA E3 ubiquitin ligase complex, which releases HY5 from its repression through ubiquitination and proteasomal degradation. The transcriptional activator HY5 induces the expression of genes such as those associated with UV-B acclimation and tolerance (biosynthesis of sunscreen metabolites, DNA repair, photoprotection, etc.). HY5 also induces genes encoding distinct proteins with regulatory roles that concurrently provide feedback regulation of UVR8 and cryptochrome photoreceptor activities (note that the control of cry1 activity by UV-B through UVR8 signalling remains to be demonstrated). BIC1 and BIC2 are thought to mainly block cry1 homodimerization, whereas RUP1 and RUP2 are thought to primarily facilitate UVR8 re-dimerization (although additionally blocking UVR8 monomerization cannot be excluded).

Cellular signal transduction is usually composed of positive regulatory factors that function as “accelerators” as well as their specific negative feedback regulators that function as “brakes”. Indeed, plant photoreceptors are activated upon photon absorption, which in conjunction with negative feedback regulation establishes a photoequilibrium according to the prevailing light conditions. For example, cryptochromes form an equilibrium between active homodimeric and inactive monomeric states, whereas UVR8 equilibrates between an active monomeric and an inactive homodimeric state^6,17,34,35^. Thus, both types of receptors are regulated at the level of their oligomeric state, with UVR8 “photomonomerizing” and cryptochromes “photodimerizing”. Moreover, both cryptochromes and UVR8 activate expression of their own negative feedback regulators, namely BIC1/BIC2 and RUP1/RUP2, respectively^13,17^. These regulatory proteins interact directly with the respective photoreceptors and facilitate their reversion to the ground state and/or inhibit their activation^13,14,17,18,34,36^ (Fig. 6). Interestingly, *BIC1* and *BIC2* are strongly induced by UVR8 signalling, although BIC1 and BIC2 apparently do not directly function in UV-B responses. Reciprocally, *RUP1* and *RUP2* are strongly induced by cryptochrome signalling, although RUP1 and RUP2 do not function in responses to monochromatic blue light^13^. We show here that the UVR8 photoequilibrium is indeed responsive to cryptochrome-mediated blue-light signalling, thus identifying a novel photoreceptor co-action mechanism to balance UV-B sensitivity of plants under the polychromatic spectrum of sunlight (Fig. 6).

Phytochromes also mediate *BIC1*/*BIC2* and *RUP1*/*RUP2* transcriptional activation in response to red and far-red light^13,18^, suggesting that phytochromes contribute to the cross-regulation of cryptochromes and UVR8 through activation of their negative feedback regulators. However, the effect of phytochrome-mediated red light signalling on RUP1/RUP2 induction and UVR8 re-dimerization is very moderate if compared to the effect of blue light (Supplementary Fig. 5). In phyB signalling, positive feedback regulation is mediated by the phyB-interacting proteins PHOTOPERIODIC CONTROL OF HYPOCOTYL 1 (PCH1) and PCH1 LIKE (PCHL), which suppress phyB dark reversion from the active Pfr to the inactive Pr state^37,38^. Interestingly, in addition to red light, *PCH1* and *PCHL* are transcriptionally activated by blue light and UV-B^5,37^. Indeed, blue light was shown to increase the response to red light facilitated by phyB^37^. Notwithstanding the physiological effects, the cross-regulation of these photoreceptor regulators is likely associated with COP1 inactivation and activation of HY5, which is the common signalling node of cryptochromes, phytochromes, and UVR8^5,9,10,20–23,34,39,40^. The convergence of these three wavelength-specific signalling pathways on COP1-HY5 is likely the basis for their overlapping photomorphogenic responses, which include hypocotyl growth inhibition, phenylpropanoid accumulation, flowering, and gene expression changes^1–3,20,34,41^. In particular, as demonstrated here, phytochrome, cryptochrome, and UVR8 signalling all contribute to establishing UV-B tolerance, suggesting that plants evolved photoreceptor cross-regulation mechanisms to globally balance their light responses in nature.

In contrast to the single mutants, *cry1 uvr8* and *phyB uvr8* double mutants were not viable under UV-B in our growth chamber experiments, demonstrating functional redundancies between cry1 and UVR8 as well as between phyB and UVR8. Interestingly, however, the *cry1 phyB* double mutant could tolerate UV-B exposure, suggesting a major role for UVR8 even in the absence of both phyB and cry1. The potential contributions of the additional cryptochrome (cry2) and four phytochromes (phyA and phyC–E) remain to be determined. Moreover, phototropin 2 (phot2) plays a major role in chloroplast avoidance in response to high light^42,43^. As UV-B levels are high on sunny days and UV-B affects the photosynthetic machinery in chloroplasts^44,45^, a contribution of phot2 to UV-B tolerance under natural conditions is to be anticipated. Independent of the probable roles for additional photoreceptors in UV-B tolerance, recent work showed that triple-mutant plants lacking cry1, cry2, and UVR8 did not survive under natural sunlight, whereas they survived well when UV-B was specifically filtered out^25^. Thus, cryptochromes and UVR8 are indeed required for survival under natural conditions.

It is of note that our data provide evidence that the UVR8 signalling pathway is hyperactive in *cry1 cry2* mutants and BIC1/BIC2 overexpression lines. In contrast, the absence of enhanced RUP1 and RUP2 induction and associated lack of a clear effect on UVR8 activity in *bic1 bic2* indicates that endogenous BIC1 and BIC2 do not play a major role in inhibiting cry activation under the conditions employed, including developmental stage, fluence rate, and kinetics. Notwithstanding this, the hyperactivity of UVR8 signalling in *cry1 cry2* suggests that enhanced UVR8 signalling contributes to UV-B tolerance in the cryptochrome mutants. As red light also induces *RUP1* and *RUP2* expression, although to an apparently much lower level, a similar argument can be made for enhanced UVR8 signalling in phyB mutants. Moreover, as UVR8 signalling induces *BIC1* and *BIC2* expression, enhanced cryptochrome signalling in *uvr8* single mutants may contribute to their UV-B tolerance; however, such a response remains to be shown. Thus, interactions within the network of phytochrome, cryptochrome, and UVR8 signalling pathways contribute to UV-B tolerance, allowing plant survival in sunlight in the natural environment. These cross-pathway interactions involve the commonly targeted COP1 E3 ubiquitin ligase and the upregulation of other photoreceptor-specific inhibitors. An exact understanding of these interactions and the individual contributions of the 13 Arabidopsis photoreceptors will provide a full appreciation of the roles photoreceptors play in plant survival. It is evident that RUP1 and RUP2 present a node for the integration of different light qualities allowing cross-regulation of the UVR8 activity state, which thus adapts UV-B tolerance strategies to the ambient light environment. It can also be speculated that other signalling pathways triggered by environmental signals beyond light perception may affect RUP1 and RUP2, thereby providing further cross-regulation of UVR8-mediated photomorphogenesis and UV-B acclimation.

## Methods

### Plant material, growth conditions, and UV-B irradiation

*Arabidopsis thaliana* mutants *hy5-215*^46^, *uvr8-6*^5^, *rup1-1, rup2-1, rup1-1 rup2-1*^13^, *cry1-304, cry2-1, cry1-304 cry2-1*^47^, *phyA-211*^48^, *cry1-304 cry2-1 phyA-412*^49^, *phyB-9, phyA-211 phyB-9*^37^, *bic1-1 bic2-1*^17^, as well as *bic1D-1* (Col/*Pro*_*35S*_*:BIC1*) and overexpression lines *Pro*_*35S*_*:BIC1-GFP#18*, *Pro*_*35S*_*:BIC2-GFP#47*^17^, and *cry1-304/Pro*_*35S*_*:GFP-CRY1*^50^ are all in the Columbia (Col) accession. Mutants *uvr8-1*^51^, *hy4-2.23N*^52^, and *phyB-5*^53^ are in the Landsberg *erecta* (Ler) accession. *hy4-3*^54^, *uvr8-7*^5^, and *uvr8-7/Pro*_*35S*_*:YFP-UVR8*^55^ are in the Wassilewskija (Ws) accession.

Arabidopsis seeds were surface sterilized with sodium hypochlorite and sown on half-strength Murashige and Skoog basal salt medium (MS; Duchefa) containing 1% (w/v) agar (Applichem) supplemented with 1% (w/v) of sucrose, except for in those experiments involving etiolated seedlings. After 2-d stratification at 4 °C, seedlings were grown in white light or seeds were exposed to 6-h white light to induce germination before being grown in darkness at 22 °C.

For light-response experiments, light-emitting diode (LED) chambers (CLF floraLEDs, CLF Plant Climatics) were used (blue light: 50 μmol m^−2^ s^−1^, peak 470 nm; red light: 50, 100 and 150 μmol m^−2^ s^−1^, peak 670 nm; half-bandwidth of 20 nm). White-light fluorescent tubes Osram L18W/30 (3.6 μmol m^−2^ s^−1^) were used in addition to supplemental UV-B from Philips TL20W/01RS narrowband UV-B tubes (0.015 and 0.06 mW cm^−2^) or not^29^.

For UV-B tolerance assays, plants were grown on soil with a 16-h day/8-h night cycle (22 *°*C/18 °C) in GroBanks (CLF Plant Climatics) with Philips Master TL-D 58 W/840 white-light fluorescent tubes (120 μmol m^−2^ s^−1^), supplemented or not with UV-B from Philips TL40W/01RS narrowband UV-B tubes (0.07 mW cm^−2^)^41^.

### Quantitative real-time PCR

Plant total RNA was isolated with the Plant RNeasy kit including DNase treatment according to the manufacturer’s instructions (Qiagen). Synthesis of cDNA was performed using the TaqMan Reverse Transcription Reagents kit according to the manufacturer’s standard protocol (Thermo Fisher Scientific). Each quantitative real-time PCR (qRT-PCR) reaction contained cDNA synthesized with a 1:1 mixture of oligo(dT) primers and random hexamers from 300 ng of total RNA. qRT-PCR was performed using a PowerUp SYBR Green Master Mix (Thermo Fisher Scientific). *PP2AA3* (*PROTEIN PHOSPHATASE 2A SUBUNIT A3*) was used as a reference gene^56^. The primers for *RUP1*, RUP1_qRT_fw (5′-AAG TGC CTG TTT CCG AGA GA-3′) and RUP1_qRT_rv (5′-GTG GAT CCC ACA TTT GAA CC-3′), and for *RUP2*, RUP2_qRT_fw (5′-TTG TGG ATC GGA AAA CAA CA-3′) and RUP2_qRT_rv (5′-CAC TGG TCC ACA CCT GAT TG-3′) were used to quantify mRNA accumulation of *RUP1* and *RUP2*, respectively. The others primers for qRT-PCR in this work were published as follows: *BIC1* and *BIC2*^17^, *HY5* and *CHS*^57^, *DREB2A*^58^, and *ELIP2*^59^.

### Immunoblot analysis

For analysis of UVR8 dimers, proteins were extracted in 50 mM Tris, pH 7.6, 150 mM NaCl, 2 mM ethylenediaminetetraacetic acid (EDTA), 10% (v/v) glycerol, 5 mM MgCl_2_, 1% (v/v) Igepal (Sigma), 1% (v/v) protease inhibitor mixture for plant extracts (Sigma), and 10 μM MG132. Twenty micrograms of proteins was separated by electrophoresis in 10% (wt/v) SDS–polyacrylamide gels and gels were UV-B-irradiated before electrophoretic transfer to PVDF membrane^6,14^.

For anti-RUP2 and anti-actin immunoblot analysis, total proteins were extracted in 50 mM Na-phosphate (pH 7.4), 150 mM NaCl, 10% (v/v) glycerol, 5 mM EDTA, 1 mM dithiothreitol (DTT), 0.1% (v/v) Triton X-100, 50 μM MG132, 2 mM Na_3_VO_4_, 2 mM NaF, and 1% (v/v) protease inhibitor mixture for plant extracts (P9599; Sigma-Aldrich). Protein concentration was determined using the Bio-Rad Protein Assay Dye Reagent Concentrate according to the manufacturer’s instructions. Proteins were separated by 12% SDS-PAGE and transferred to nitrocellulose membranes. Anti-RUP2^(1−15)^ (1:500 dilution)^41^, anti-UVR8^(426−440)^ (1:4000)^5^, and anti-actin (1:20,000) (A0480, Sigma-Aldrich) were used as the primary antibodies. Horseradish peroxidase (HRP)-conjugated anti-rabbit and anti-mouse immunoglobulins (Dako A/S) were used as the secondary antibodies (1:20,000). Immunodetection was performed using an ECL Plus Western Detection Kit and revealed with an ImageQuant LAS 4000 mini CCD camera system (GE Healthcare). For quantitative analysis of UVR8 re-dimerization, immunoblot membranes were probed with IRDye 800 fluorescent dye-coupled anti-rabbit secondary antibodies (1:10,000) and analysed using the Odyssey® IR imaging system (LI-COR Biosciences). ImageJ was used for quantifications, for which bands intensities for UVR8 dimers and UVR8 monomers were added to represent 100%. Data are presented as percentage of UVR8^dimer^ amounts (UVR8^dimer^/UVR8^total^).

### Hypocotyl length measurements

Four-day-old Arabidopsis seedlings were grown under continuous irradiation in a light field with Osram L18W/30 tubes (3.6 µmol m^−2^ s^−1^; measured with a LI-250 Light Meter; LI-COR Biosciences) supplemented with Philips TL20W/01RS narrowband UV-B tubes. The UV-B range was modulated by the use of 3-mm transmission cut-off filters of the WG series with half-maximal transmission at 305 nm (WG305, +UV-B at 0.06 mW cm^−^^2^; measured with a VLX-3W UV Light Meter equipped with a CX-312 sensor; Vilber Lourmat), at 320 nm (WG320, +UV-B at 0.015 mW cm^−^^2^), or 345 nm (WG345, −UV-B) (Schott Glaswerke)^5,29^. Seedlings were scanned and their hypocotyl lengths were measured (*N* = 30) using ImageJ software. Experiments were independently repeated three times.

### Anthocyanin extraction and measurement

Plant seedlings were grown 4 d in the same conditions as for hypocotyl length measurement, and 50 mg plant material was homogenized for 10 s using a Silamat S5 mixer (Ivoclar Vivadent). 250 μl of acidic methanol (1% HCl) was added to each sample and placed in an overhead shaker at 4 °C for 1 h. Samples were centrifuged for 1 min at 14,000 rpm and the supernatant was used to quantify anthocyanin content based on the absorption at 535 and 650 nm. Values were reported as A_535_ − 0.25 (A_650_) g^−1^ fresh weight.

### Statistical analyses

Statistical analyses were performed using GraphPad Software Prism 8 software (San Diego, California). Statistical significance of the differences between means was determined using Student’s *t* tests (protein quantification data) or one-way ANOVA and post-hoc Tukey tests (hypocotyl length data).

## Supplementary information

Supplementary Information

## Data Availability

The data supporting the findings in this study are available and described within the paper and its supplementary files. The source data underlying Figs. 1–5 and Supplementary Figs. 1−5 are provided as a separate Source Data file. All biological material generated in this study are available from the corresponding author upon reasonable request.

## Source data

Source Data
