# Supplementary material for: Cryptochrome-mediated blue-light signalling modulates UVR8 photoreceptor activity and contributes to UV-B tolerance in Arabidopsis
Source: Nat Commun. 2020 Mar 12;11:1323. doi: 10.1038/s41467-020-15133-y (PMC7067804; doi:10.1038/s41467-020-15133-y)
Supplement: Supplementary file 1 — Supplementary Information [file 41467_2020_15133_MOESM1_ESM.pdf]

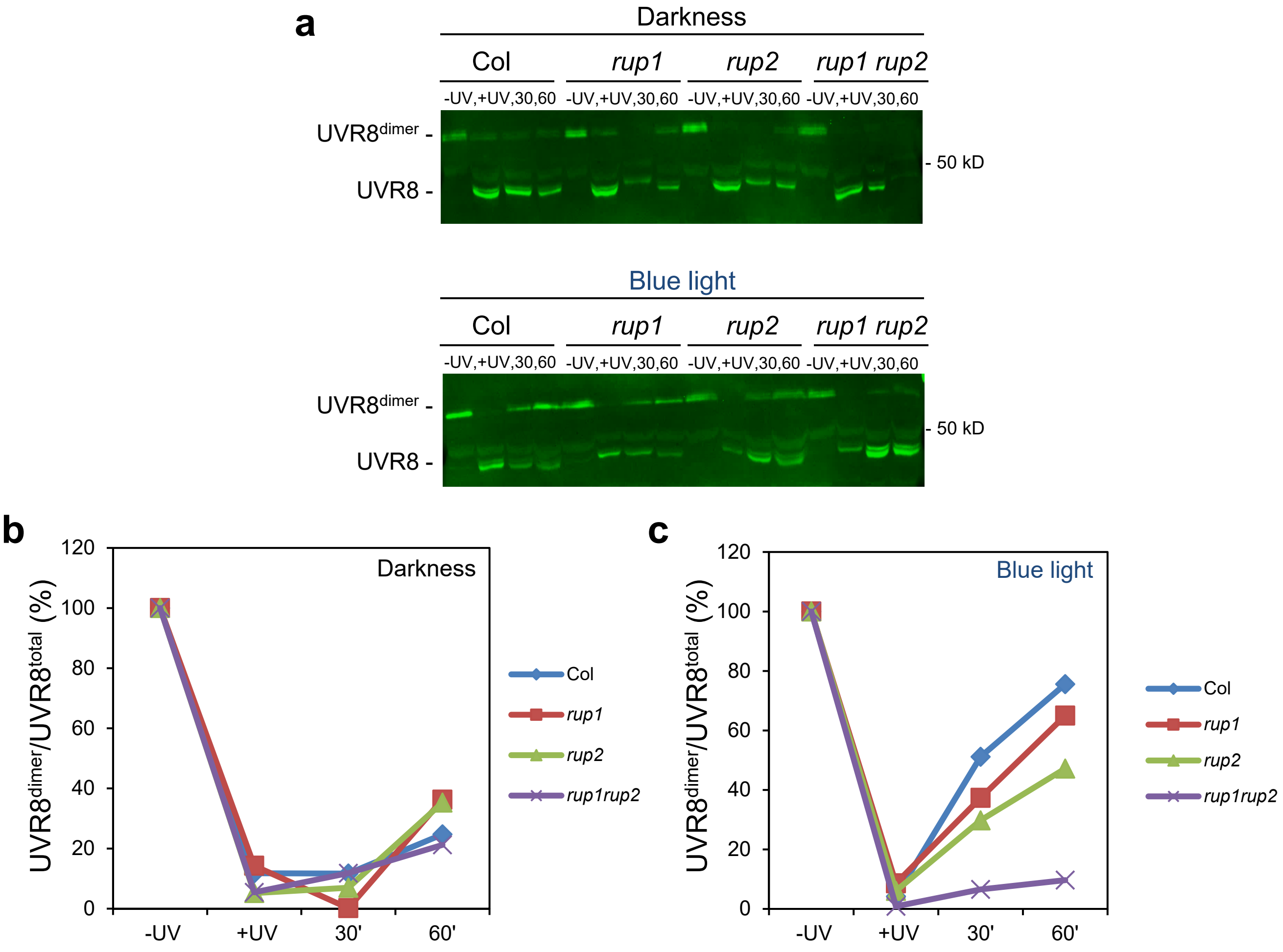

Supplementary Fig. 1 RUP1 and RUP2 play redundant roles in blue-light enhancement of UVR8 re-dimerization. **a** Immunoblot analysis of UVR8 homodimer and monomer levels (non-heat-denatured samples) in wild-type (Col), *rup1-1* (*rup1*), *rup2-1* (*rup2*) and *rup1-1 rup2-1* (*rup1 rup2*) seedlings immediately prior to UV-B treatment (-UV), immediately following 15-min broadband UV-B treatment, and post-UV-B treatment following 30- and 60-min recovery in darkness (30 and 60, respectively). Seedlings were either pre-treated for 12 h with blue light before the UV-B treatment (Blue light; lower panel) or not (Darkness; upper panel). **b**, **c** Quantification of the $UVR8_{dimer}/UVR8_{total}$ ratio (%) in a representative experiment.

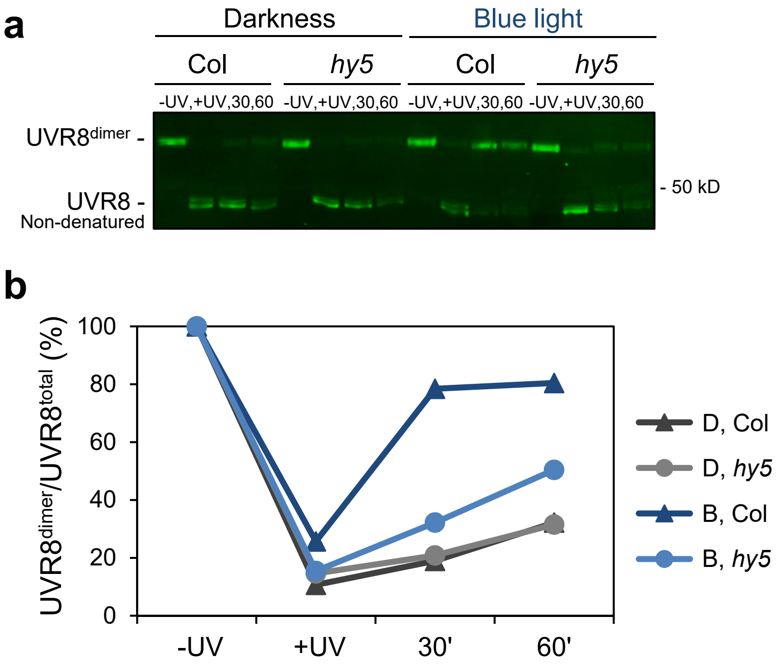

Supplementary Fig. 2 HY5 is required for the blue light-mediated repression of UVR8 activity. **a** Immunoblot analysis of UVR8 re-dimerization in wild-type (Col) and *hy5-215* (*hy5*) seedlings. UVR8 dimers were detectable in non-heat-denatured protein samples. Parallel denatured samples demonstrated equal amounts of UVR8 protein. The time points for sampling (-UV, +UV, 30' and 60') were as schematically shown in Fig. 2a. **b** Quantification of the $UVR8^{dimer}/UVR8^{total}$ ratio (%) in response to UV-B in Col and *hy5* seedlings pre-treated with blue light (B) or not (D).

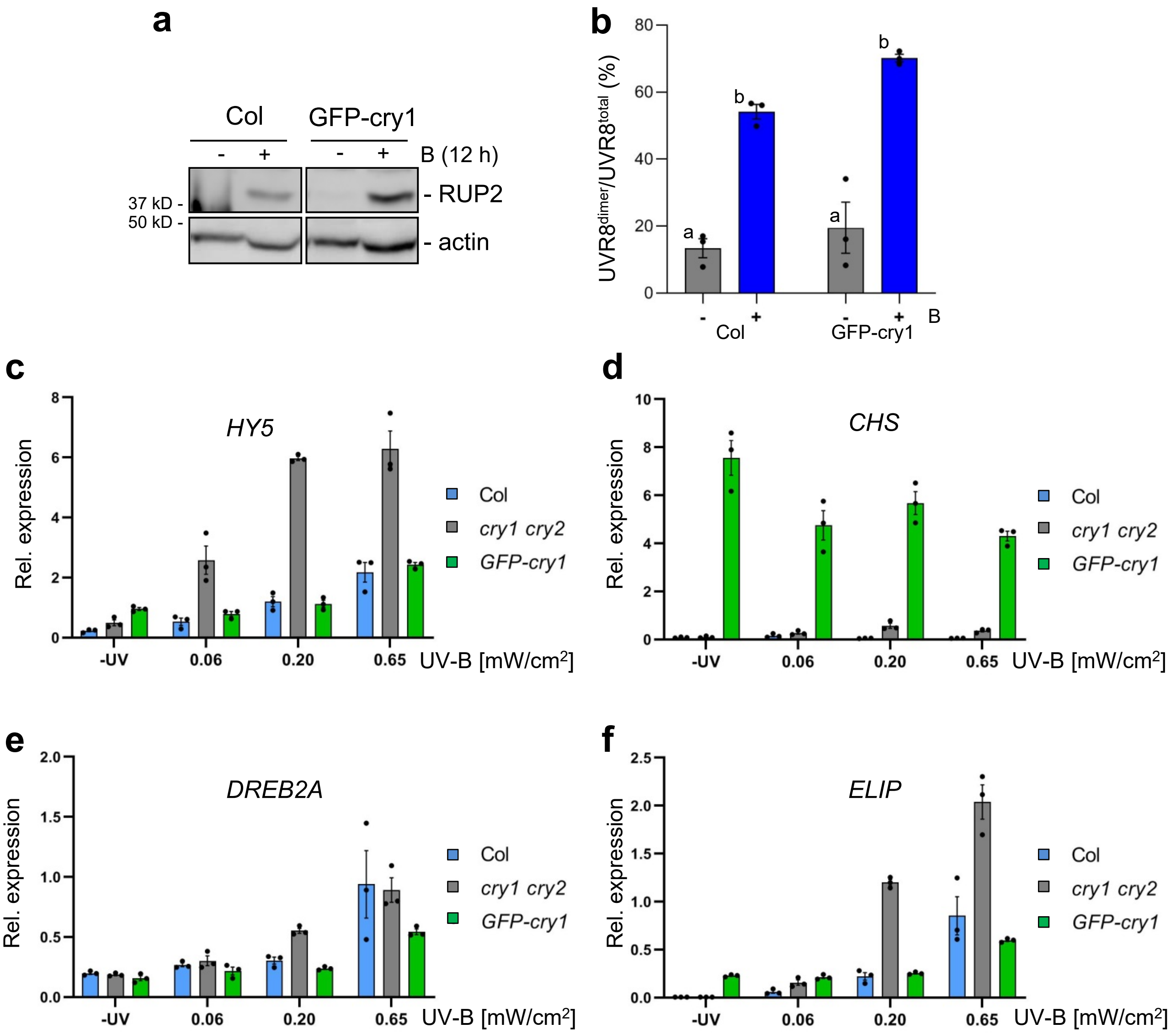

Supplementary Fig. 3 GFP-cry1 overexpression enhances blue light-induced RUP2 accumulation and UVR8 re-dimerization. **a** Immunoblot analysis of RUP2 protein level in 4-d-old wild type (Col) and a GFP-cry1 overexpression line (*cry1*/*Pro*$_{35S}$*:GFP-CRY1*) grown in darkness and exposed to 12 h of blue light (+) or not (-). Actin is shown as protein loading control. **b** Quantification of the UVR8^{dimer}/UVR8^{total} ratio (%) after a 30 min recovery post-UV-B treatment in wild type (Col), *cry1* (*cry1-304*), and GFP-cry1 seedlings pre-treated with 12-h blue light or not (darkness). Data are means ± SEM (*N* = 3). Shared letters indicate no statistically significant difference in the means (P > 0.05; one-way ANOVA followed by post-hoc Tukey test). **c-f** Seven-d-old seedling grown under 12-h light /12-h dark cycles were irradiated for 15 min with broadband UV-B at different intensities (0.06, 0.20, and 0.65 mW cm-2) or not (-UV). qRT-PCR analysis of (**c**) *HY5*, (**d**) *CHS*, (**e**) *DREB2A* and (**f**) *ELIP2* in Col ,*cry1 cry2* and *cry1* over expressing line (*cry1/35s::GFP-cry1*) seedlings grown as per the schematic in panel (Fig. 4e). Data are means ± SEM (*N* = 3).

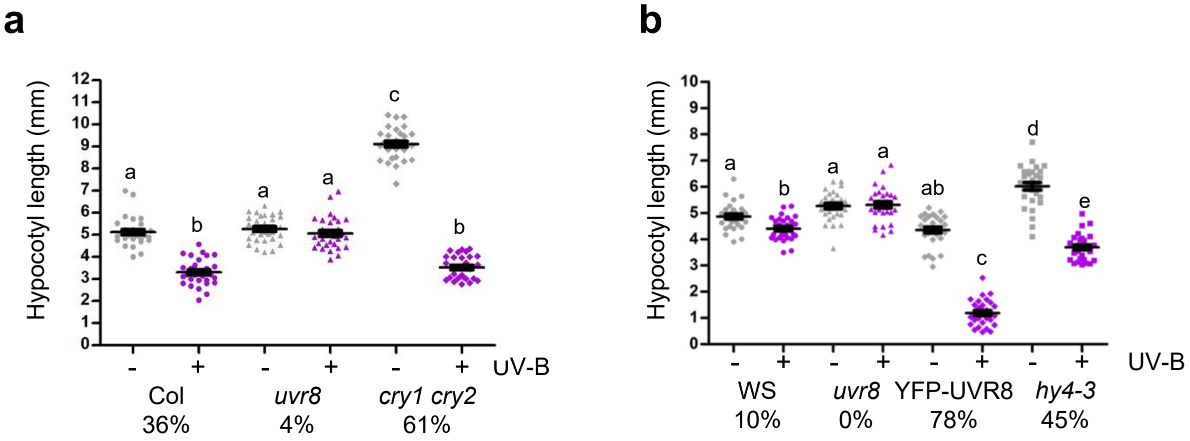

Supplementary Fig. 4 cry1 mutants show enhanced UV-B-induced hypocotyl growth inhibition. **a** Quantification of hypocotyl length in 4-d-old wild type (Col), *uvr8-6* (*uvr8*) and *cry1-304 cry2-1* (*cry1 cry2*) seedlings grown in white light (3.6 μmol m^{-2} s^{-1}) supplemented with UV-B (+UV-B; 0.06 mW/cm^2) or not (-UV-B). Data are means ± SEM (*N* = 30). Numbers below bars show the relative hypocotyl growth inhibition by UV-B as a percentage. **b** Quantification of hypocotyl length in 4-d-old wild type (Ws), *uvr8-7* (*uvr8*), *uvr8-7/35S::GFP-UVR8* (*GFP-UVR8*) and *hy4-3* (*cry1*) seedlings grown in white light (3.6 μmol m^{-2} s^{-1}) supplemented with UV-B (+UV-B; 0.015 mW/cm^2) or not (-UV-B). Data are means ± SEM (*N* = 30). Shared letters indicate no statistically significant difference in the means (P > 0.05; one-way ANOVA followed by post-hoc Tukey test).

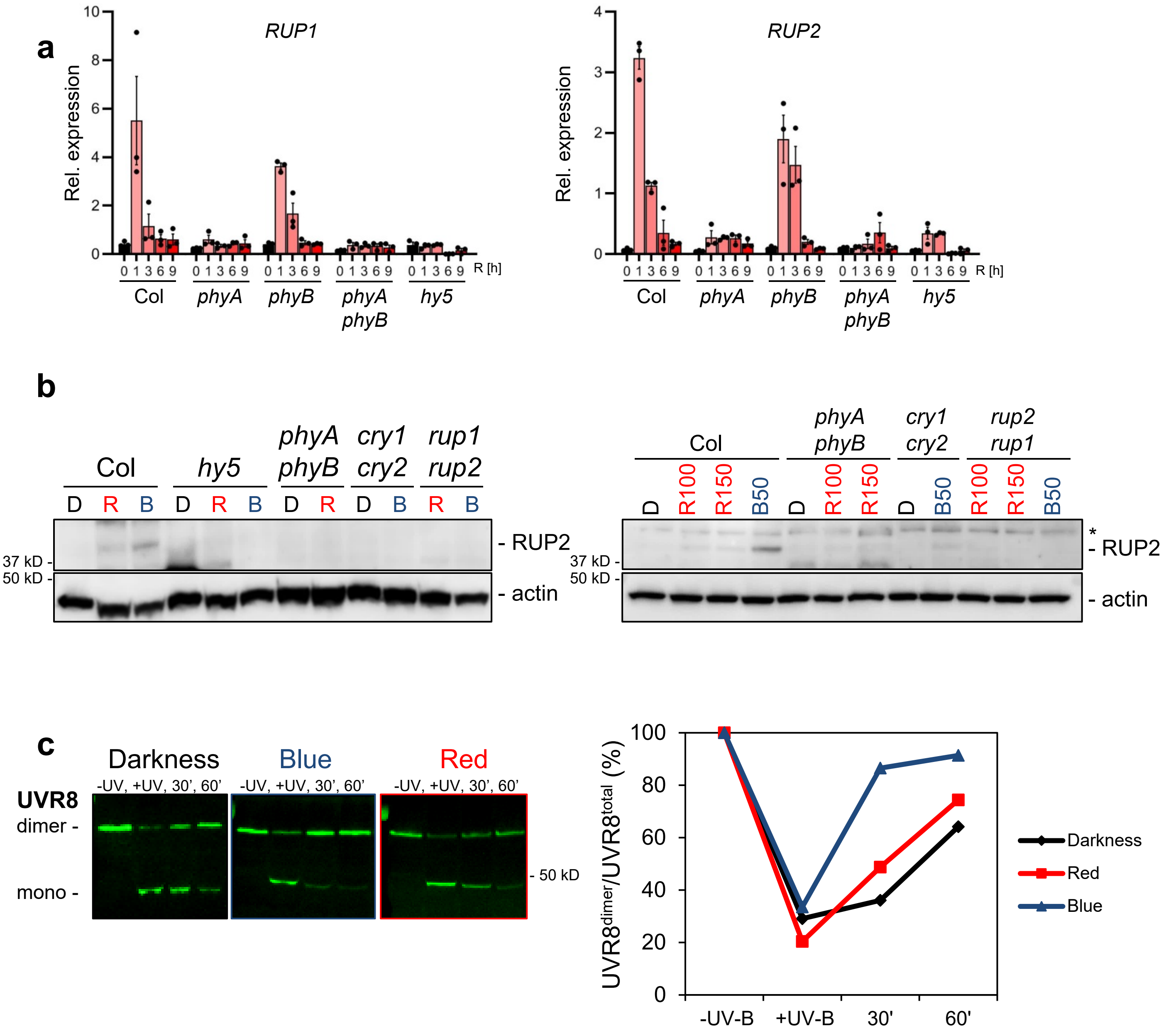

Supplementary Fig. 5 Red light induction of RUP1 and RUP2 expression, RUP2 protein accumulation, and moderate enhancement of UVR8 re-dimerization. **a** qRT-PCR analysis of *RUP1* and *RUP2* expression in 4-d-old wild-type (Col), *phyA-211* (*phyA*), *phyB-9* (*phyB*), *phyA-211 phyB-9* (*phyA phyB*) and *hy5-215* (*hy5*) seedlings grown in darkness or treated with red light (R; 50 µmol m^{-2} s^{-1}) for 1, 3, 6, or 9 h. Data are means ± SEM (N = 3). **b** Immunoblot analysis of RUP2 protein level in 4-d-old Col, *hy5-215* (*hy5*) *phyA-211 phyB-9* (*phyA phyB*) *cry1-304 cry2-1* (*cry1 cry2*) and *rup1-1 rup2-1* (*rup1 rup2*) seedlings grown in darkness and exposed to 12 h of 50 µmol m^{-2} s^{-1} blue light (B) or red light (R) or not (D) in the left panel. Same genotypes seedlings grown in darkness and exposed to 12 h of blue light (Blue 50; 50 µmol m^{-2} s^{-1}), red light (Red; 100 and 150 µmol m^{-2} s^{-1}) or not (Darkness) in the right panel. Actin is shown as protein loading control. **c** Quantification of the $UVR8^{dimer}/UVR8^{total}$ ratio (%) in response to UV-B in Col seedlings pre-treated with blue light or red light or not (Darkness).
